# Hydrogel-Forming Microneedle Arrays for Enhanced Transdermal Drug Delivery

**DOI:** 10.1002/adfm.201200864

**Published:** 2012-07-09

**Authors:** Ryan F Donnelly, Thakur Raghu Raj Singh, Martin J Garland, Katarzyna Migalska, Rita Majithiya, Cian M McCrudden, Prashant Laxman Kole, Tuan Mazlelaa Tuan Mahmood, Helen O McCarthy, A David Woolfson

**Affiliations:** School of Pharmacy, Queens University Belfast97 Lisburn Road, Belfast BT9 7BL, UK

## Abstract

Unique microneedle arrays prepared from crosslinked polymers, which contain no drug themselves, are described. They rapidly take up skin interstitial fluid upon skin insertion to form continuous, unblockable, hydrogel conduits from attached patch-type drug reservoirs to the dermal microcirculation. Importantly, such microneedles, which can be fabricated in a wide range of patch sizes and microneedle geometries, can be easily sterilized, resist hole closure while in place, and are removed completely intact from the skin. Delivery of macromolecules is no longer limited to what can be loaded into the microneedles themselves and transdermal drug delivery is now controlled by the crosslink density of the hydrogel system rather than the stratum corneum, while electrically modulated delivery is also a unique feature. This technology has the potential to overcome the limitations of conventional microneedle designs and greatly increase the range of the type of drug that is deliverable transdermally, with ensuing benefits for industry, healthcare providers and, ultimately, patients.

## 1. Introduction

**Figure 1:**
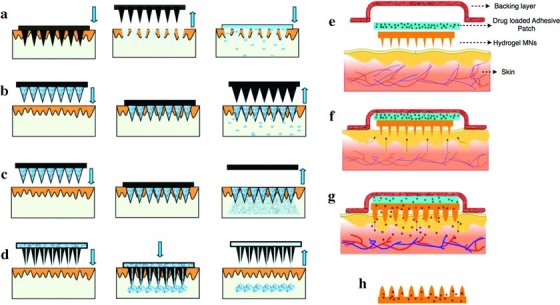
Schematic representation of methods of MN application to the skin to achieve enhanced transdermal drug delivery. a–d) Traditional methods of MN-mediated drug delivery across skin. (a) uses solid MN that are applied and removed to create transient micropores, followed by application of a traditional transdermal patch. (b) uses solid MN coated with drug for instant delivery. MN areremoved after coating material dissolves. (c) uses soluble polymeric/carbohydrate MN containing drug that dissolve in skin interstitial fluid over time, thereby delivering the drug. (d) uses hollow MN for delivery of fluids containing drug. e–h) Our novel hydrogel-forming polymeric MN for controlled transdermal drug delivery. (e) shows an exploded view of our novel integrated hydrogel MN patch, which consists of a backing layer, a drug-loaded adhesive patch and a solid crosslinked hydrogel MN array. (f) shows application of the integrated hydrogel MN patch to the skin surface. This is followed by diffusion of water into the crosslinked integrated MN patch. (g) shows diffusion of water, which causes controlled swelling of the MN arrays, forming an in situ hydrogel conduit. This further results in liberation and diffusion of drug molecules from the adhesive patch through the hydrogel MN into the skin. (h) shows that the hydrogel MN arrays remain intact, even after removal from the skin, thereby leaving no polymeric material in the skin following drug delivery.

Microneedle arrays (MN) are minimally-invasive devices that painlessly by-pass the skin's *stratum corneum,* which is the principal barrier to topically-applied drugs.^1^ MN (50–900 μm in height, up to 2000 MN cm^−2^) have been extensively investigated in recent years as a means to enhance transdermal drug and vaccine delivery.^2^–^10^ No MN array-based drug delivery systems are presently marketed, due to the relatively recent developments that have made such devices a reality. Indeed, the subsequently rapid progression of the field has inevitably led to some difficulties: silicon, the most commonly-employed MN material to date, has dubious biocompatibility and broken silicon or metal MN could cause skin problems;^5^ solid, non-coated MN require a two-step application process, which is undesirable for patient use^5^, ^6^ (**Figure** **1**a); accurately coating MN is difficult and these coated MN subsequently only deliver a very small amount of drug as a bolus^2^–^4^, ^6^ (Figure 1b); MN-induced holes normally close over very quickly (<1 h). The rate of hole closure can be slowed by heavy occlusion, but cannot be prevented;^7^ biomolecules can be significantly degraded by the heating used to produce polymeric MN from molten polymers or carbohydrates^2^–^4^, ^8^ (Figure 1c); and hollow MN have only one outlet and can become blocked by compressed dermal tissue.^2^–^4^ (Figure 1d).

Recently, MN loaded with drug have been prepared under ambient processing conditions from aqueous gels based on FDA-approved polymers.^9^ Such systems, which dissolve in skin interstitial fluid to release their drug payload, have overcome many of the above difficulties. However, while transdermal delivery of small (<500 daltons) water-soluble molecules from both MN and baseplate is possible with such systems, only macromolecules (e.g., insulin) located in the MN themselves are deliverable.^10^ In addition, while the polymers employed for fabricating these MN have long-established safety profiles, they have never before been administered intradermally. Regulatory authorities will inevitably require extensive studies investigating routes of metabolism and elimination, possibly delaying commercialisation. Since MN materials will encounter viable skin cells, unlike conventional transdermal and topical drug delivery systems, regulators may also require MN systems to be sterile. Here we describe unique MN arrays prepared from crosslinked polymers which contain no drug themselves, as shown in Figures 1e–h. Instead, they rapidly take up skin interstitial fluid upon skin insertion to form continuous, unblockable, hydrogel conduits from patch-type drug reservoirs to the dermal microcirculation (Figure 1f,g). Importantly, such MN, which can be fabricated in a wide range of patch sizes and MN geometries, can be easily sterilised, resist hole closure while in place and are removed completely intact from the skin (Figure 1h). Delivery of macromolecules is no longer limited to what can be loaded into the MN themselves and transdermal drug delivery is now controlled by the crosslink density of the hydrogel system rather than the *stratum corneum,* while electrically modulated delivery is also a unique feature.

## 2. Materials and Methods

This study comprehensively investigated the utility of our novel hydrogel-forming MN arrays in transdermal drug delivery. Mechanical properties, in vitro/in vivo permeation of model drug substances, combined effect with iontophoresis (ITP), biocompatibility, and safety in human subjects were investigated. The methods for each study are detailed below.

### 2.1. Chemicals

Poly(methylvinylether/maelic anhydride) (Gantrez AN-139) was provided by ISP, Guildford, UK. Poly(ethyleneglycol) 10,000 daltons and all drug substances were obtained from Sigma-Aldrich, Poole, Dorset, UK. All other chemicals were of analytical reagent grade.

### 2.2. Preparation and Mechanical Testing of MN

Aqueous blends containing 15% w/w poly(methylvinylether/maelic acid) (PMVE/MA) and 7.5% w/w poly(ethyleneglycol) 10,000 (PEG) were utilized to fabricate MN by using laser-engineered silicone micromould templates.^10^, ^15^, ^16^ Optimum polymeric composition was determined in our previous hydrogel work.^11^–^13^ MN were crosslinked (esterification reaction) by heating at 80 °C for 24 h.^11^–^13^ Mechanical properties of these hydrogel MN (with 600 μm height, 300 μm width at base, 150 μm interspacing, and 3 × 3 arrays) were determined as reported previously.^8^, ^10^, ^15^, ^16^

### 2.3. In Vitro Permeation Studies

**Figure 2:**
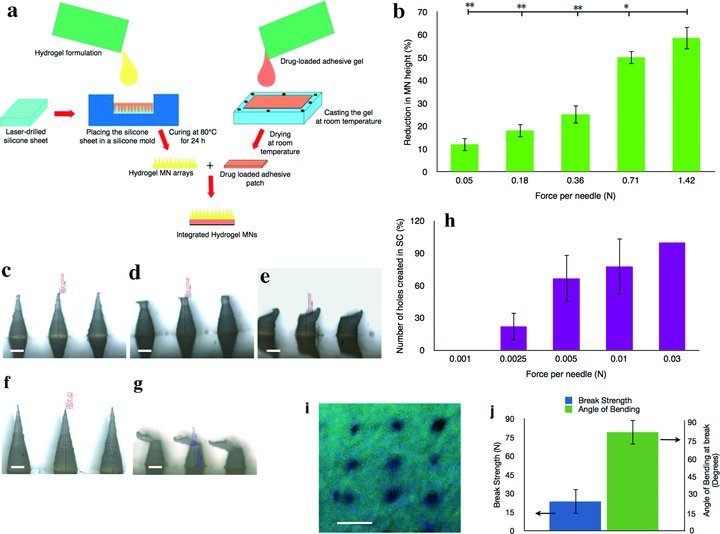
Fabrication and mechanical properties of novel hydrogel MN arrays. a) The method of fabrication of the integrated hydrogel-forming MN system from aqueous polymeric blends using custom laser-engineered silicone micromoulds. b) Percentage reduction in height of hydrogel-forming MN following axial load, Means ± S.D, n = 45. c–e) Digital microscopic images of hydrogel-forming MN following axial compression; where c) MN were subjected to a force of 0.05 N per needle, d) MN were subjected to a force of 0.36 N per needle, and e) MN were subjected to a force of 1.42 N per needle. f,g) Digital microscope images of hydrogel-forming MN subjected to transverse forces; f) before application and g) after application of 0.51 N per needle. h) A histogram depicting the percentage number of holes created in the *stratum corneum* of neonatal porcine skin in vitro following application of a range of insertion forces. i) A digital image of methylene blue staining showing 100% MN penetration of the *stratum corneum* of neonatal porcine skin in vitro, following application of an insertion force of 0.03 N per needle or greater. j) Forces required to break the MN base-plates and the angle of bending of the MN base-plates at break, Means ± SD, n = 6. The scale bar in (i) represents 200 μm. *P < 0.05 and ** *P* < 0.001. Error bars in b, h, and j indicate standard deviations.

The ability of the novel hydrogel-forming MN in enhancing and controlling transdermal drug delivery was investigated by using six hydrophilic solute molecules of increasing molecular weight: fluorescein-isothiocyanate labelled bovine serum albumin (FITC-BSA), insulin, methylene blue (MB), caffeine (CF), theophylline (TP), and metronidazole (MZT) with molecular weights of ≍67,000, ≍6000, 319.85, 194.19, 180.17, 171.15 Daltons, respectively. For studies involving high-molecular weight molecules, a pore-forming agent (NaHCO_3_) was added to the hydrogel formulation. Adhesive patches, as stated below, containing the above molecules at defined loadings, were then attached to the upper baseplates of hydrogel-forming MN, with the novel composite system termed as “integrated MN” (**Figure** **2**a). Permeation was then investigated from this integrated MN system and compared with that of the adhesive patches alone.

Adhesive patches were prepared, by using a casting method, from aqueous blends of 10% w/w PMVE/MA and 5% w/w tripropyleneglycol methyl ether (TPM).^28^ Required amounts of solute molecules were either added directly into the aqueous blends or dissolved in 0.01 M HCl prior to addition to the aqueous blends. An aliquot (2.7 g) of aqueous blend was cast into silicone moulds (30 × 30 mm^2^) and dried under a gentle air flow for 48 h. Attaching the formed adhesive patches with gentle pressure to hydrogel-forming MN yielded the integrated MN system.

Transdermal permeation was investigated in vitro across dermatomed neonatal porcine skin (300–400 μm thickness, previously shown by us to be the optimum skin model for prediction of in vivo performance of dissolving polymeric MN)^29^ by using a modified Franz-cell setup, as described previously.^10^, ^15^, ^16^ Integrated MN were applied to the dermatomed skin by using a custom spring-activated applicator^16^ at a force of 11.0 N per array. Adhesive patches were applied using gentle pressure. Sample volumes of 300 μL were withdrawn, in triplicates, and analysed by the methods below. The release medium for FTIC-BSA was 0.05% w/w SDS in phosphate buffered saline (PBS) at pH 7.4, for insulin 0.1 M Tris buffer at pH 10 (due to stability issues) and for the other drug molecules PBS at pH 7.4 was used.

### 2.4. Analysis of Drug Permeation

Receptor medium samples for insulin, TP and MZT were analysed using validated analytical methods reported previously.^10^, ^15^, ^30^ FITC-BSA was quantified using a gradient HPLC method (Agilent Technologies 1200 Series, Stockport, UK) in which the separation was performed on a C_4_ (4.6 mm × 50 mm, 5 μm) analytical column (Symmetry 300, Waters Ireland, Dublin). The mobile phase gradient consisted of 0.1% triforoacetic acid (TFA) in acetonitrile (ACN) and 0.1% TFA aqueous solution. The gradient linearity changed from 20:80 (ACN: TFA) to 90:10 in 8 min and continued for 1.0 min at the ratio of 90:10. The reversal to the initial conditions was attained within 1.0 min, followed by a 3.0 min re-equilibration period. The total analysis run time was 13.0 min. The injection volume was 20 μl and elution flow rate was 1.0 ml min^−1^. FITC-BSA was detected using a fluorescence detector with excitation and emission wavelengths set at 490 nm and 520 nm, respectively. Data were processed using Agilent Rapid Res software. CAF was analyzed following modification of a literature method.^31^ Briefly, an isocratic HPLC method was used with a reverse phase C-18 (Waters Spherisorb 5 μm ODS 4.6 × 150 mm, Waters Ireland, Dublin) analytical column. The column was thermostated at 35 °C. The mobile phase consisted of 90% of 0.52% glacial acetic acid and 10% of 50% ACN: 50% tetrahydrofuran. The flow rate was 1.0 ml min^−1^. Sample injection volume was 50 μl. UV detection was performed at 273 nm. Data were processed using Agilent Rapid Res software. MB was analysed at 664 nm using a UV microplate reader (Powerwave XS, Bio-Tek Instruments Inc., Minooski, USA).

### 2.5. In Vitro ITP Studies

We further investigated the combined effect of integrated MN and ITP on in vitro permeation of TP, MB, fluorescein sodium (FS), insulin and FITC-BSA. In this case, TP, MB and FS were loaded at a concentration of 3 mg cm^−2^ in the adhesive patches. FITC-BSA and insulin were loaded at concentration of 2.5 and 5 mg cm^−2^, respectively. The methodology for this study is similar to that stated above. For studies involving application of an electrical current, an Ag electrode was used as the anode and an Ag-AgCl electrode used as the cathode. The delivery electrode was placed directly on top of the integrated MN, whilst the return electrode was placed into the receiver medium via the side arm of the Franz cell. A commercially available power supply (Phoresor II, Iomed, Lake City, FL, USA) was used to deliver a current of 0.5 mA for a period of 6 h for in vitro studies. A sample volume of 300 μl was collected at predetermined time intervals and was assayed by the analytical methods described above. For samples containing FS, UV-spectroscopy was used at a λ_max_ of 497 nm.

### 2.6. In Vivo Studies

Delivery of insulin, FITC-BSA and MZT from the integrated MN (with 600 μm height, 300 μm width at base, 50 μm interspacing, & 19 × 19 arrays) was investigated in a rat model. For studies involving insulin, diabetic Sprague-Dawley rats were selected. The methodology was similar to that in our previous publication, which involved soluble MN.^10^ Positive controls were performed by subcutaneously injecting bovine insulin solution in PBS pH 7.4 at a dose of 0.2 IU per animal. Integrated MN based on adhesive patches containing 5 mg cm^−2^ insulin were applied to the shaved skin (Gillette Mach 3 Power, Procter & Gamble, Hampshire, UK) on the rats' backs by using the spring-activated applicator at a force of 11.0 N per array. Skin integrity was confirmed using transepidermal water loss measurements made using a Deflin Vapometer (Deflin Technologies Kuopio, Finland) Following application, the integrated MN were held in place for 12 h with the help of a self-adhesive silicone sheet.

Blood samples were collected at pre-defined time intervals over a 12 h period by lateral tail vein prick and blood glucose levels (BGL) were measured using a glucometer (Accu-Check Aviva, Roche Ltd., Mannheim, Germany). Blood glucose levels were expressed as the percentage of initial BGL and calculated values were plotted against time to obtain blood glucose level-time profiles. C_max_, denoting maximum% decrease in BGL, was calculated by subtracting the lowest% BGL from 100, as described elsewhere.^32^ T_max_, denoting time required to achieve C_max_, was also reported.

Integrated MN containing FITC-BSA at 2.5 mg cm^−2^ loading in patches, were applied to healthy rats in an analogous way to insulin-loaded patches. A 0.25 ml aliquot of blood was drawn from the tail vein and collected into heparinised tubes (Microvette CB300, Sarstedt, Leicester, UK) at pre-defined time intervals over a period of 24 h. Plasma samples were obtained by centrifuging the collected blood samples at 10 000 rpm for 10 min. All samples were analysed, within 2.0 h of collection using spectrofluorimetry at an excitation wavelength of 490 nm and emission wavelength of 520 nm.

In vivo delivery of MZT from the integrated MNs was also investigated in rat models. The concentration of MZT versus time data obtained using the dried blood spot (DBS) method reported previously^30^ was subjected to non-compartmental analysis (WinNonlin version 2.1, Pharsight, USA) to determine pharmacokinetic parameters, namely, area under curve (AUC), C_max_ and T_max_. The steady state blood concentration (C_ss_) of MZT after application of integrated MNs was calculated by using the equation:


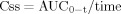


For in vivo studies, involving application of an electrical current, a circular PVC ring (diameter 2.0 cm) was secured onto the rats back at the site of integrated hydrogel MN or adhesive patch application, using cyanoacrylate glue (Loctite Ltd, Dublin Ireland). The Ag electrode anode was then placed on top of the integrated MN or adhesive patch and held in place using a layer of double-sided adhesive tape (Henkel Ltd, Cheshire, UK) and an additional layer of Micropore tape (3M Ireland, Dublin). A second PVC ring (diameter 2.0 cm) was then secured at an adjacent site, ≍ 2 cm away from the site of MN application. An aliquot of PBS pH 7.4 (1.0 ml) was then placed into the centre of this second PVC ring and an Ag-AgCl electrode was attached at the site using Micropore tape. The Phoresor™ II was used to deliver a current of 0.5 mA for a 2 h period after which the integrated MN and/or adhesive insulin/FITC-BSA patch were removed.

All animal experiments throughout this study were conducted according to the policy of the Federation of European Laboratory Animal Science Associations (FELASA) and The European Convention for the protection of vertebrate Animals used for Experimental and Other Scientific Purposes, with implementation of the principle of the 3Rs (replacement, reduction, refinement).

### 2.7. Biocompatability Evaluation

Biocompatability of PEG-crosslinked PMVE/MA-based hydrogel MN materials was performed in three different cell-lines using an indirect test with two suspended cell-lines; fibroblasts (Balb/3T3) and keratinocytes (NRERT-1)^24^ and a 3D keratinocyte organotypic raft culture. In all three cases, an MTT [3-(4,5-dimethylthiazol-2-yl)-2,5-diphenyl tetrazolium bromide] assay was used to determine any cytotoxic effects.

To depict conditions pertaining to actual in vivo application of MN, a Franz-cell setup was used to expose only the MN and not the baseplate to the extraction medium. A Silescol membrane was sandwiched between the receptor and donor compartment of the Franz-cells and the hydrogel-forming MN were applied in a similar manner as stated above. The receptor compartment was filled with 12.0 ml of DMEM (Dulbecco's modified Eagle's medium) containing 4.5 g L^−1^ glucose, 2.0 mM L-glutamine, 10% foetal calf serum, 1.0 IU ml^−1^ penicillin and 1.0 μg ml^−1^ streptomycin. After 24 h of application, the DMEM solution was collected and sterile filtered. The medium (100%, 50% and 10% diluted in fresh DMEM) was exposed to cell monolayers of Balb3T3 and NRERT-1 keratinocytes at 1 × 10^4^ cells ml^−1^ in the 96-well culture plate for 24 hrs. Following 24 h incubation at 37 °C, the test extracts were assessed for cell viability by means of the quantitative MTT assay at 540 nm (Biotrak II Plate Reader, Amersham Biosciences, Cambridge, UK). A total of six replicates were studied for each extract, including control (sterile DMEM media) in the same plate. In 3D keratinocyte organotypic raft type culture studies, the 3D keratinocyte tissues were pre-incubated (at 37 °C and 5% CO_2_) for 1.0 h in assay medium before starting the experiment. Prior to dosing with MN extract the assay medium was renewed. An aliquot (50 μL) MN extract (undiluted) was applied atop the tissues in triplicate and incubated for 24.0 h. At the end of the exposure period, the tissue cultures were gently rinsed with PBS, incubated in fresh medium for a further 24.0 h and finally placed into plates containing 0.5 mg ml^−1^ of MTT solution at 1.0 ml cm^−2^ cultured tissue (4.0 ml). Following 3.0 h incubation and a rinse with PBS, the reduced MTT was extracted by submerging the tissues in 2.0 ml of isopropanol and shaking for 2.0 h. The absorbance of the extraction solutions was again measured at 540 nm. The mean absorbance was calculated for each tested sample (undiluted MN extract). The mean result of the negative control (H_2_O) tissues was set to represent 100% viability.

### 2.8. In Vitro 3D Skin Irritancy Test

In vitro skin irritancy tests were performed by using a 3D reconstructed human skin model (EpiSkin, Skin Ethic Laboratories, Lyon, France). The assay endpoint for irritancy measurements was IL-1α (Interleukin-1 alpha). An aliquot (10 μl) of sterile-filtered hydrogel extract, obtained as described above, was exposed to the reconstructed epidermis of the EpiSkin skin model. A negative (PBS) and positive (5% w/v sodium dodecyl sulphate, SDS) control were also included in the protocol. In each case, the epidermis of EpiSkin skin model was exposed at room temperature, according to the manufacturer's recommendations. Following exposure for 24 h, EpiSkin™ constructs were washed with PBS, and transferred to wells that contained fresh maintenance medium, and allowed to recover for 42 h at 37 °C, 5% CO_2_. The IL-1α content of the recovery medium was assessed using a human IL-1α specific ELISA (Thermo Scientific/Pierce, IL, USA).

### 2.9. Human Volunteer Studies

In this study, gamma-sterilised hydrogel-forming MN were applied to the ventral forearm skin of six healthy human volunteers (3 men and 3 women, aged between 23 and 31 years), with no pre-existing skin conditions. They were asked not to wash or apply any cosmetic formulations on the ventral forearm during the study period. The School of Pharmacy's Ethical Committee, Queen's University Belfast, approved this study.

Hydrogel-forming MN arrays were attached to waterproof plasters (Elastoplast, Beiresdorf, Hamburg, Germany) using double-sided adhesive tape. The spring loaded applicator was used to apply the MN-containing plasters Each volunteer was subjected to four interventions, a control plaster (without MN), a plaster containing MN baseplate only, a plaster containing hydrogel-forming MN (11 × 11 array) and a plaster containing hydrogel MN (19 × 19 array), as shown.

The study was conducted at a controlled room temperature of 20 °C and a relative humidity of 45 ± 5%. The subjects were acclimatized in this room for 15 min prior to the start of the measurements. This study involved the application of waterproof plasters, as described above, to the ventral forearm on three different occasions; 1) Patches applied and removed immediately (i.e., 0 h treatment group); 2) Patches applied and removed after 2 h (i.e., 2 h treatment group), and 3) Patches applied and removed after 24 h (i.e., 24 h treatment group).

Before application of the patches the ventral forearm were cleansed with a sterile swab (Boots Pharmaceuticals, Nottingham, UK) in each case. Four square areas (≍1.5 cm^2^) were marked on the ventral forearm of each subject using a ballpoint pen. The square areas were located at equidistance on each forearm. Before the application of the plasters, TEWL and clinical photographs of skin were recorded, as described below. Following application of the plasters, the applicator was activated against each plaster to ensure consistent MN penetration in each volunteer (confirmed using OCT, as described previously).^16^ Immediately upon application, and after removal of the plasters (i.e., in 0, 2 and 24 h treatment groups), the VAS, TEWL and any change in skin colour was recorded in all the four interventions, as follows;

### 2.10. Visual Analogue Scale (VAS) Pain Scores

Pain sensation associated with plasters was determined on a VAS. The pain intensity rating (i.e., VAS) was measured immediately after plaster application and immediately upon removal. The VAS was determined by asking the volunteers to mark on a 10 cm line, anchored by word descriptors at each end (“No Pain = 0 cm” and the other end the “Worst Pain Imaginable = 10 cm). The volunteers mark on the line the point that he or she feels represents their perception of their current state. The VAS score is determined by measuring in centimeters from the left hand end of the line to the point that the volunteer marks.^33^

### 2.11. Transepidermal Water Loss

TEWL was measured to determine the level of disruption to skin barrier function following application of the plasters. The Vapometer was used to measure TEWL at a control site (previously marked with squares of ≍1.5 cm^2^ areas) pre- and post-application of plasters. TEWL measurements have been established as a routine method for evaluating the integrity of skin, which has been subjected to either physical or chemical treatment.^34^, ^35^ TEWL measurements were taken by carefully resting the TEWL probe horizontally on the application site, with the probe head vertical and perpendicular to the skin. TEWL measurements recorded for a period up to 4 hours following patch removal

### 2.12. Clinical Scoring

Clinical scoring was used to determine irritation threshold in all study volunteers. Clinical scores were based on visual inspection following guidelines of the International Contact Dermatitis (ICD) Research Group and the North American Contact Dermatitis Group. Clinical photographs of the skin before and after plaster application were captured using a Nikon D40X digital camera equipped with a Nikon AF-S VR Micro-Nikkor 105 mm f/2.8G IF-ED lens and a Nikon Close-up Speedlight Commander Kit R1C1 with two wireless remote Speedlight SB-R200 flash units (Nikon UK Ltd, Kingston, UK) mounted on a ring on either side of the lens and using aperture priority. The camera was fixed on a tripod and was adjusted to know height so that the distance between the ventral forearm and the camera lens were consistent throughout the study. Images were then printed on high quality photographic paper and were evaluated and scored blindly by two-experienced dermatologists. A clinical score for each test site was assigned, using the ICD scores. ICD scores were rated as 0, 0.5, 1, 2 or 3, which represent either: a negative, a barely perceptible macular erythema, a mild erythema, a moderate-intense uniform erythema or an intense erythema, vesiculation or erosion, respectively.^36^, ^37^ Optical coherence tomographic (OCT) studies, which allowed measurement of depth of MN insertion and permitted study of in-skin swelling, were conducted as described previously.^16^

### 2.13. Statistical Analysis

Physical characterisation data and in vitro drug permeation experiments were analysed using a One-Way Analysis of Variance (ANOVA), with Tukey's HSD post-hoc test. Comparisons for animal studies, cell culture work and human volunteer investigations were made using the Kruskal-Wallis tests and the Mann-Whitney U-test. In all cases *p* < 0.05 denoted significance.

## 3. Results and Discussion

### 3.1. Mechanical Characterisation

We studied the mechanical properties of MN prepared from a hydrogel-forming formulation selected as a result of our previous work with such materials.^11^–^13^ Here we report on five mechanical tests; axial and transverse deformation, skin penetration, MN base plate deformation and break. Increase in axial forces caused a progressive reduction in MN height, shown in Figure 2b. The mean percentage reduction in MN height was 12.12, 18.18, 25.21, 50.21 and 58.65%, at applied forces of 0.05, 0.18, 0.36, 0.71 and 1.42 N per needle, respectively. Figure 2c–e indicate that, even at the highest axial force, complete MN failure was not observed, indicating residual viscoelasticity, despite the relatively high glass-transition temperature (T_g_) of this material (55.82 ± 0.97 °C). Application of forces at right angles to the axes of the MN caused progressive reductions in MN height by bending. For example, at forces of ≍0.06 and 0.51 N per needle, the percentage reductions in MN height were ≍24 and 57%, respectively. Figure 2f,g shows representative digital images of MN after transverse force application. It was observed that insertion forces as low as 0.03 N per needle resulted in 100% of our MN penetrating the *stratum corneum* of neonatal porcine skin (thickness 400 μm) in vitro, as evidenced by methylene blue staining in Figure 2i. In contrast, Figure 2j shows the force required to break the hydrogel MN base-plates was approximately 23.55 N. At break point, the MN baseplate had bent to an angel of approximately 79.28°. This reflects not only the considerable strength of the material, but also its conformability, important since, on a micron-scale, the skin is not perfectly flat. Hence, MN arrays must ideally have a degree of flexibility in their baseplate to ensure all MN penetrate the skin when applied. While this is true for the system described here, the same cannot be said for silicon, where we have seen that hard brittle baseplates can fracture upon application.

### 3.2. In Vitro Release Studies

For successful passive transdermal transport, molecules should ideally have intermediate Log P (1–3) values and molecular weights less than 500 Daltons.^1^, ^14^ A wide range of techniques aimed at bypassing the *stratum corneum* barrier have successfully overcome these limitations. However, tape-stripping, sonophoresis, electroporation and heat/laser ablation are all much less practical than MN technology, which has been widely acclaimed for its potential to realise considerable patient benefit in the near future. Recently, we have demonstrated a significant enhancement in transdermal insulin transport in vitro using our dissolving poly(methylvinylether-co-maleic acid) (PMVE/MA) MN.^10^ While percutaneous administration of these insulin-loaded MN arrays to diabetic rats resulted in a dose-dependent hypoglycaemic effect, sustained administration was limited by the fact that only the insulin contained in the needles themselves was released. This is an acknowledged problem for macromolecule-loaded dissolving polymeric MN. Since this is the type of MN system that currently shows the most promise and due to the ever-increasing number of macromolecular drugs with delivery problems, solutions are urgently required. In the present study, we have for the first time, demonstrated MN-mediated sustained percutaneous delivery of both low and high molecular weight compounds.

**Figure 3:**
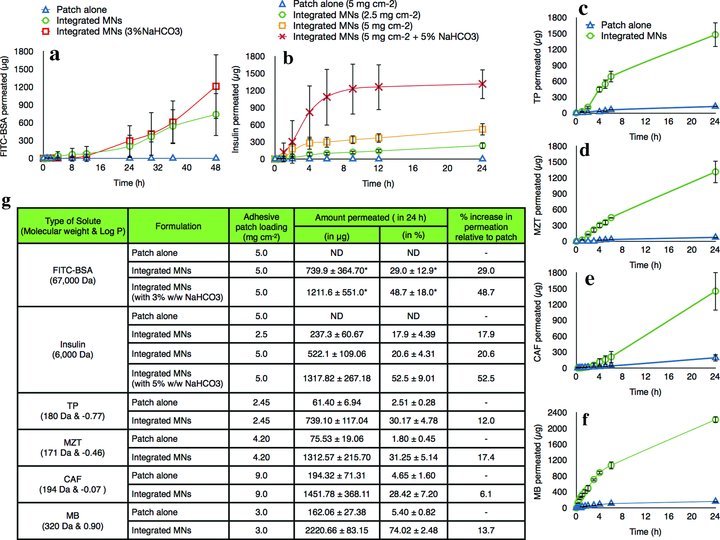
In vitro cumulative permeation results of different drug molecules delivered from integrated hydrogel-forming MN across dermatomed neonatal porcine skin of 300 ± 50 μm thickness. a) In vitro permeation of FITC-BSA at a loading of 5 mg cm^−2^ from adhesive patches, integrated MN and integrated MN prepared from aqueous blends containing 3% w/w of NaHCO_3_. b) In vitro permeation of insulin from adhesive patches alone (at a loading of 5 mg cm^−2^), from integrated MN (at a loading of 2.5 mg cm^−2^), from integrated MN (at a loading of 5 mg cm^−2^) and from integrated MN prepared from aqueous blends containing 5% w/w of NaHCO_3_ (at a loading of 5 mg cm^−2^). c) In vitro permeation of TP at a loading of 2.45 mg cm^−2^ from adhesive patches and integrated MN. d) In vitro permeation of MZT at a loading of 4.20 mg cm^−2^ from adhesive patches and integrated MNs. e) In vitro permeation of CAF at a loading of 9 mg cm^−2^ from adhesive patches and integrated MN. f) In vitro permeation of MB at a loading of 3 mg cm^−2^ from adhesive patches and integrated MN. g) Table indicating the amount of each solute molecule released, in both μg and percentages, at the end of 24 h study period. Means ± SD, n = 4, * indicates permeation observed after 48 h. ND in (g) indicates not determined.

When drug-loaded patches were attached to drug-free hydrogel-forming MN and the integrated devices applied to dermatomed neonatal porcine skin in vitro, controlled drug administration was achieved, with rates and extents of permeation independent of molecular weight and Log P values (**Figure** **3**). The demonstrated ability to deliver relatively small water soluble molecules in milligram per square centimeter amounts in a sustained fashion is likely to be of great value to industry and, ultimately, patients. Most conventional drugs are small water soluble molecules and many treatment regimes would benefit from the enhanced bioavailability and reduced dosing frequency that transdermal delivery offers. The biotechnology boom continues to produce peptide and protein drugs with great potential. However, most of these exciting new drugs must be dosed parenterally, due to their high molecular weights, extreme hydrophilicities and propensity for gastrointestinal destruction. Here we have shown sustained transdermal transport of a peptide and protein in milligram per square centimeter amounts over a 24 hour period. Importantly, we have previously demonstrated that the nature of this hydrogel formulation can easily be altered on a case-by-case basis to improve delivery,^11^–^13^ suggesting utility in delivery of a range of macromolecules.

### 3.3. Combined Effect of ITP and Integrated MN

MN have been widely hailed as exciting alternatives to parenteral injection, complete with enhanced ease of use and reduced potential for transmission of infection. However, MN-based delivery typically relies on relatively slow drug diffusion through MN-induced holes in the *stratum corneum* or dissolution of drug-loaded polymeric MN or drug coatings on silicon or metal MN. This is in stark contrast to the rapid delivery possible using conventional needles and syringes. It has recently been proposed that the combination of skin barrier impairment using MN coupled with iontophoresis (ITP) may allow rapid delivery of vaccines or hormones (e.g., insulin), as well as enabling delivery of conventional drug substances to be precisely controlled.^17^, ^18^ Ultimately, this may enable bolus, pulsatile or responsive drug administration. Combination of MN and ITP has shown to lead to a synergistic enhancement in transdermal delivery of a range of molecules.^17^–^21^ However, the necessity for a two-stage application process (MN applied and removed, then ITP applied) when using this approach may be cumbersome for clinicians and patients, particularly given the importance of ensuring that the drug-loaded formulation is placed correctly over the microporated skin area. Secondly, it is known that the process of skin closure begins almost immediately upon MN removal, unless the skin area is kept under heavy occlusion.^7^ This effectively limits the time available for enhanced drug delivery. Such problems may be overcome by the amalgamation of ITP with in situ polymeric MN delivery systems. As such, the present study evaluated the potential for the combination of our novel hydrogel MN devices and ITP.

**Figure 4:**
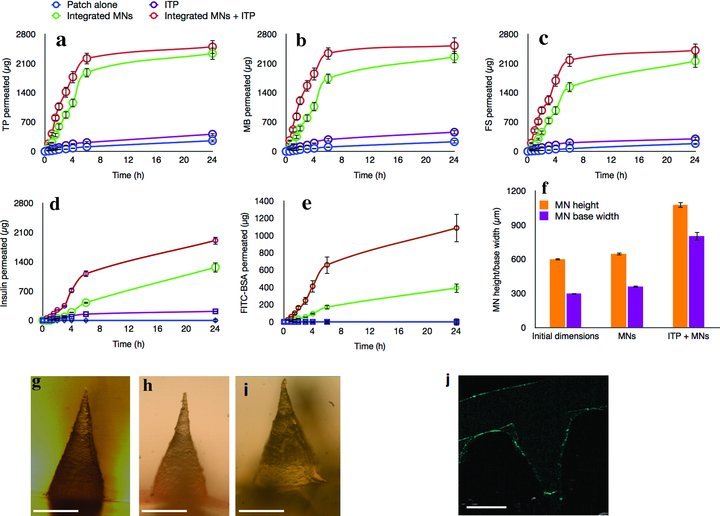
Combined effect of ITP and integrated hydrogel-forming MN on in vitro permeation of different drug molecules across dermatomed neonatal porcine skin of 300 ± 50 μm thickness and the effect of ITP on in-skin MN swelling. a–e) Cumulative permeation of TP, MB, FS, insulin and FITC-BSA, respectively. f) OCT assessment of changes in MN dimensions following insertion into rat skin for a period of 1 h ex vivo. g–i) Representative light microscopy images of hydrogel-forming MN; g before skin insertion, h) 1 h after insertion into skin and i) 1 h after insertion into skin and application of a continuous electric current of 0.5mA. j) A representative OCT image of a hydrogel-forming MN array following insertion into rat skin ex vivo and application of an electric current of 0.5 mA for a period of 1 h. Scale bars represent a length of 300 μm. Error bars in (a–f,h) indicate standard deviations.

**Figure** **4**a–e illustrates the combined effect of ITP and integrated hydrogel MN on permeation of five different solutes across dermatomed neonatal porcine skin. In general, the combination of ITP with the novel hydrogel MN array led to a greater rate and overall extent of transdermal delivery, in comparison to integrated MN alone. However, significant enhancements were only observed for the biomolecules insulin and FITC-BSA. This was particularly notable for FITC-BSA and is understandable, given the approximate 12,000 Dalton cut-off associated with conventional ITP.^1^, ^19^

In an additional ex vivo experiment, optical coherence tomographic (OCT) analysis revealed that application of an electrical current leads to a dramatic increase in the swelling of the hydrogel-forming MN. It can be seen in Figure 4f–i that, 1 h after MN insertion into rat skin, MN height increased from ≍610 to 650 μm, whilst the MN base width increased from ≍307 μm to 360 μm. However, following application of an electrical current for 1 h, MN height increased to ≍1076 μm (p < 0.001), and the MN base width to approximately 802 μm (p < 0.001). This equates to an approximate 5.75-fold increase in MN surface area, in comparison to integrated hydrogel MN swelling under passive conditions. Such pronounced dimensional changes, most likely due to enhanced water uptake by electoosmosis as we have previously postulated for the PEG-crosslinked PMVE/MA system,^13^ may partially explain the enhancements in delivery observed. The ability of these MN to imbibe skin interstitial fluid is likely to find use in minimally-invasive patient monitoring, since drug concentrations in skin interstitial fluid frequently reflect those in plasma.^22^ This would overcome many of the problems associated with direct blood sampling.

### 3.4. In Vivo Studies

**Figure 5:**
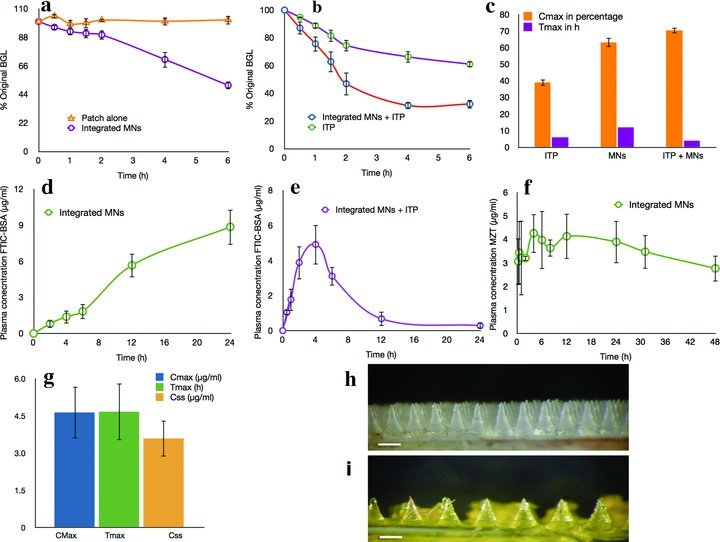
In vivo permeation studies in a rat model. a)% drop in blood glucose levels following delivery of insulin from patches alone and from integrated hydrogel-forming MN. b) The% drop in blood glucose levels following application of an electric current (0.5 mA for 2 h) with the insulin-loaded patch and in combination with integrated hydrogel-forming MN. c) C_max_ and T_max_ values of insulin for the different delivery strategies shown in (a,b) investigated within the diabetic rat model in vivo. d) Plasma concentration of FITC-BSA delivered from integrated MN patches - no detectable FITC-BSA was delivered from the patches alone. e) Plasma concentration of FITC-BSA delivered from integrated MN patches combined with ITP. f) The in vivo pharmacokinetic profile of MTZ following delivery by integrated hydrogel-forming MN. g) The pharmacokinetic parameters (C_max_, T_max_ and C_ss_) of MZT in rats. h–i) Representative light microscopy images demonstrating that hydrogel-forming MN arrays remain intact; h) before insertion and i) after removal from a rat in an in vivo experiment after a 12 h application/insertion period. Scale bars represent a length of 300 μm. Error bars in (a–f) indicate standard deviations.

In order to demonstrate the utility and advantages of our novel hydrogel MN technology, we carried out a range of in vivo rat experiments (**Figure** **5**a–f). Application of integrated hydrogel MN arrays resulted in a controlled reduction in blood glucose levels (BGL) in diabetic rats. BGL dropped to ≍90% of its original level within 2 h and fell further to ≍37% by the end of the 12 h experimental period. Anodal ITP alone (applied for a 2 h period, after which the electrodes and insulin-loaded adhesive patch were removed) resulted in a maximal drop in BGL to ≍61% of its original BGL value within 6 h, with BGL returning to normal by 12 h. The combination of integrated hydrogel MN and anodal ITP (applied for a 2 h period, after which the electrodes and MN/insulin patch were removed) led to a rapid reduction in BGL, dropping to ≍47% within 2 h and ≍32% within 6 h, before returning to normal values by 12 h. It was observed that the combined effect of integrated hydrogel MNs and ITP showed a significantly greater C_max_ (maximum% decrease in BGL value) (70.42 ± 1.86%) than ITP alone (39.06 ± 2.10%) (*p* < 0.001) or MN alone (63.16 ± 2.82%) (*p* < 0.001). Furthermore, the time taken to reach this value was significantly reduced through the combination of integrated MN and ITP (4 h), in comparison to ITP (6 h) (*p* < 0.001) or MN alone (12 h) (*p* < 0.001).

Sustained transdermal delivery of the high-molecular weight protein FTIC-BSA was clearly observed following application of integrated MN, with peak plasma concentrations reaching 8.86 ± 1.49 μg ml^−1^ at 24 h. The combination of integrated hydrogel MN and ITP led to a significantly accelerated FTIC-BSA permeation, with detectable FTIC-BSA levels found in plasma after only 30 min. Following termination of the electric current and complete removal of the MN device after 2 h of application, FTIC-BSA plasma levels were found to be 3.89 ± 0.96 μg ml^−1^. This is ≍ 4.8 fold greater than the 0.81 ± 0.32 μg ml^−1^ detected at the same period through the use of integrated hydrogel MN alone (*p* < 0.001). It was found that the FTIC-BSA plasma levels continued to rise to peak levels of 4.92 ± 1.15 μg ml^−1^ at 4 h. In contrast, application of an adhesive FTIC-BSA-loaded patch, or the use of ITP alone, did not result detection of FTIC-BSA in plasma.

The low molecular weight hydrophilic drug metronidazole (MZT) was found to rapidly appear in plasma and maintained quite constant levels over the 48 h study period. The mean pharmacokinetic parameters of MZT, administered through the integrated MN, was determined by a non-compartmental analysis of the whole blood, where the C_max_ was 4.64 ± 1.05 μg ml^−1^ at a T_max_ of 4.67 ± 1.15 h (Figure 5g). The mean area under the curve (AUC_0–48 h_) was found to be 172.24 ± 24 μg h ml^−1^. The C_ss_ was calculated by dividing AUC_0–48 h_ with total study time (48 h) was found to be 3.59 ± 0.73 μg ml^−1^. Based on the literature, the minimum effective concentration for MZT is 6 μg ml^−1^.^23^ If MZT loading in the integrated MN was to be increased, the minimum effective concentration could easily be achieved over a prolonged period of time, negating the need for invasive intravenous infusion. One of the major advantages of the current hydrogel-based MN delivery system could be negating the need for hospitalisation, as required in infusion dosing. In the present study, MZT was used as a model drug. However, considering the potential of this new delivery system, drugs with narrow therapeutic indices, having a range of physicochemical properties (log P/log D) and/or exhibiting stability problems in infusion media could be efficiently delivered with this approach.

It was again observed that application of an electrical current led to a marked increase in the rate and extent of in-skin swelling of the hydrogel-forming MN arrays. Importantly, as with in vitro studies, the integrated hydrogel MN arrays were removed fully intact following the complete period of application in all animals, regardless of whether ITP was employed or not (Figure 5h,i). This reflects the retention of good mechanical strength by the swollen hydrogel and is likely to be of great importance as MN technology moves forwards towards commercialization, given that regulatory authorities, healthcare professionals and ultimately patients may have concerns about local or systemic reactions that may occur if portions of the needle were to break within the skin. It is our assertion that these integrated hydrogel MN are well-suited for sustained delivery of both small water soluble drugs and also higher molecular weight peptide and protein molecules. Pulsatile delivery can be achieved through application of ITP to meet on-demand requirements (e.g., delivery of insulin after a meal) or for rapid vaccine delivery.

### 3.5. Safety and Scalable Production

All drug delivery systems must not only be effective, they must also be completely safe for patient use. Accordingly, much current effort is focussed on MN prepared from FDA-approved polymeric materials, rather than silicon or metal. Such systems will also need to be shown to be safe, since administration of these polymers by the intradermal route is completely new. Sterility, or low bioburden, is likely to be a prerequisite for regulatory approval, while MN arrays must be capable of being produced in a range of sizes to suit required drug doses.

### 3.6. Biocompatability

**Figure 6:**
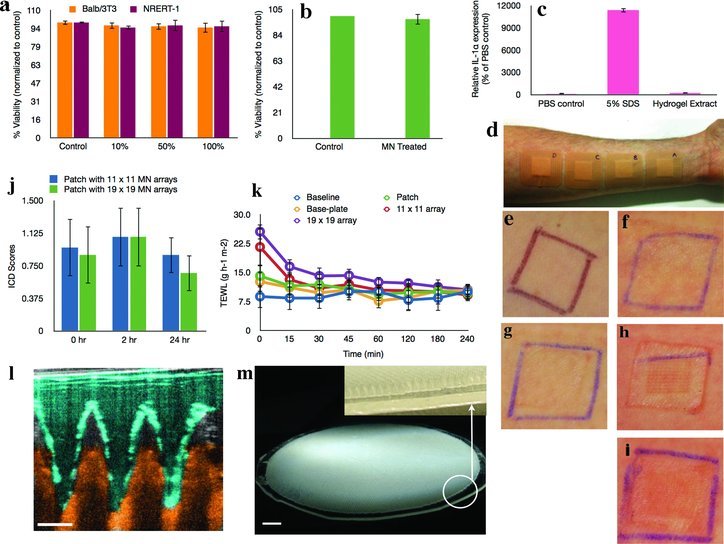
In vitro and in vivo safety studies for hydrogel-forming MN. a–c) Results of cytotoxicity studies in cell lines; a) in Balb/NRERT-1 keratinocyte cell lines; b) in 3D-keratinocyte cells lines; c) IL-1α expression by EpiSkin constructs in control conditions and on exposure to a 24 h aqueous extract of hydrogel microneedles. d) A representative pattern of application of four different plasters on the ventral forearms of human volunteers. e–i) Representative samples of clinical photographs taken to measure the ICD scores in human volunteers in a 24 h treatment group by expert dermatologists; e) before application of MN (ICD = 0); f) after removal of plaster only (ICD = 0); g) after removal of plasters containing MN baseplates only (ICD = 0); h) after removal of plasters containing 11 × 11 hydrogel-forming MN array (ICD = 0.5); i) after removal of plasters containing 19 × 19 hydrogel-forming MN array (ICD = 1). j) The mean ICD scores in human volunteers following removal of the two different densities of hydrogel-forming MN array in the 24 h treatment group. k) Mean TEWL values following removal of hydrogel-forming MN array in the 24 h treatment group. l) An OCT image 2 h following insertion of hydrogel-forming MN array (height 600 μm, width at base 300 μm, spacing 300 μm, 11 × 11 array) into human skin in vivo. m shows a digital image of hydrogel-forming MN array containing approximately 18 000 MN needles in a 25 cm^2^ area, the hydrogel needles can been seen in the inset. Error bars in (a–c,j,k) indicate standard deviations. The scale bar in (l) represents a length of 300 μm, while the scale bar in m represents a length of 5 mm.

Biocompatibility of a given material can be tested by ISO and USP standards,^24^, ^25^ which principally depend upon exposure of material to specific cells in vitro, using both direct and indirect contact approaches. Alternatively, 3D human cells cultures can also be used.^26^ In these methods, leachable products from the material will, if toxic, induce membrane damage and impairment of metabolic processes. The biocompatibility of our MN materials was evaluated in vitro using cell monolayers (Balb/3T3 and NRERT-1 keratinocytes) and a 3D skin model. **Figure** **6**a,b shows no significant reduction in cell viability in all three tests. Skin irritancy studies (Figure 6c), showed that exposure to SDS elicited a significant increase in production of the irritancy biomarker cytokine interleukin-1α (IL-1α) in a 3D skin model when compared to both control and hydrogel MN extract. These results suggest that the hydrogel-based MN material is likely to be both biocompatible and non-irritant in Figure 6c (Figure 6c).

### 3.7. Sterilization and Human Volunteer Studies

Both aseptic manufacture and post-manufacture gamma sterilization rendered MN sterile, as evidenced by the BP Sterility Test.^27^ Gamma-sterilised (^60^Co, 10 kGy) MN were applied to the ventral forearm skin of six healthy human volunteers. Application of a conventional sticking plaster only and the plaster containing the MN baseplate received a VAS score of 0 cm (i.e., no pain was felt at all) in all volunteers. However, application of plasters containing MN arrays elicited higher VAS scores, which were dependent upon the density of the MN array applied. For example, the mean VAS score associated with application of the hydrogel MN of density 361 MN per 0.5 cm^2^ (0.33 ± 0.10 cm) was greater than that for a hydrogel MN of density 121 MN per 0.5 cm^2^ (0.14 ± 0.08) (*p* < 0.001). Similarly, both hydrogel MN systems had greater recorded VAS score during wear for both the 2 h and the 24 h application groups. VAS scores were increased for both MN array designs during the 24 h treatment protocol, in comparison to the 2 h treatment protocol. Comments from volunteers regarding sensations included: “A prickly feeling noticed at times during the presence of 361 array”; “Slight pressure noticed for 361 array about 10 h after application, but not classed as pain”; “Definitely feel a sensation during 361 array application, like a scratch”; “Could feel pressure during 361 array presence, slight itchy feeling” and “For 24 h period, slight itchy feeling at night for the 361 array”.

Figure 6l shows representative cross-sectional OCT images of 121 MN arrays inserted in human skin in vivo. The images confirm that the MN punctured the *stratum corneum* barrier and the MN extended ≍ 460 μm into the skin. The widths of the MN induced holes in the *stratum corneum* were ≍265 μm in diameter. However, it is obvious that there was a clear space of ≍136 μm between the bottom of the MN baseplate and the upper surface of the *stratum corneum*, indicating that the entirety of the MN lengths were not inserted into the skin. When the 361 MN array were inserted into the skin of human volunteers, it was found that the MN extended approximately 440 μm into the skin and that the holes in the *stratum corneum* were ≍258 μm in diameter. Once again, a clear space of approximately 156 μm between the bottom of the MN baseplates and the upper surface of the *stratum corneum* was observed.

Figure 6d–i show sample clinical photographs for a 24 h treatment protocol. The irritant contact dermatitis (ICD) scores indicated no reaction with the plaster only treatment, whilst only one individual had a response graded as 0.5 for the plaster containing the MN baseplate only. The skin response following application of the MN-containing plasters was graded as only barely perceptible (0.5) or mild erythema (1), regardless of the length of time the MN array remained within the skin. Interestingly, for the 0 h and 24 h treatment protocols, the grading of the skin response following removal of the patch containing a 361 MN array was lower than that for a patch containing a 121 MN array, as shown in Figure 6j. In all cases, ICD scores of the skin site were MN arrays had been applied returned to normal (i.e., 0) within 1 h of MN removal. Furthermore, MN arrays remained intact and no damage to the MN was noted during application, wear (as evidenced by OCT, Figure 6l), or removal of MN-containing plasters.

Following removal of MN-containing plasters from the volunteers, MN dimensional changes were measured. A greater increase in MN dimensions was noted for the higher density 361 MN array in comparison to the 121 MN array (*p* < 0.001). MN height increased from 597.2 ± 7.4 to 731.2 ± 6.8 μm and MN width at base from 306.9 ± 4.5 to 379.3 ± 5.2 μm after 24 h wear for the 361 MN array. In comparison, the MN height of the 121 array increased from 599.6 ± 6.9 to 686.8 ± 7.8 μm, whilst the MN width at base increased from 305.8 ± 4.6 to 359.4 ± 5.6 μm, after residing in the skin of a human volunteer for a 24 h period. Overall, these values equate to an increase in MN surface area of 35.0%, and 51.5% for the 121 and 361 MN array designs, respectively.

Transepidermal water loss (TEWL) measurements, used worldwide to assess skin barrier function showed that the skin barrier returned to normal within 30 minutes of MN removal, regardless of the treatment protocol. Figure 6k show the results of TEWL values for the 24 h treatment group. Similar trends in TEWL values were observed in the 0 h and 2 h treatment groups.

Interestingly, we found that MN removed from skin, even after 1 min insertion, were incapable of re-insertion, due to softening by rapid imbibing of skin interstitial fluid. This is a further important safety advantage over conventional MN designs.

### 3.8. Production

In order to be commercially viable, polymeric MN must be capable of large scale production. Manufacture of arrays with areas greater than 0.5–1 cm^2^ will also be required in order to provide flexibility of dosing for drugs with different therapeutic windows. Clearly, centrifuge-based mould filling is unsuitable for production of multiple arrays or arrays with large surface areas. In addition, there are no similar dosage forms currently produced by the pharmaceutical or medical devices industries. It is our assertion that MN manufacture should resemble production of conventional transdermal patches as closely as possible. Accordingly, we used knife casting to spread aqueous PEG-containing PMVE/MA gels on laser engineered silicone moulds without sidewalls. The moulds were then placed in a vacuum chamber. This resulted in fully-formed MN arrays. The maximum patch size produced by us to date is 25 cm^2^, containing approximately 18,000 individual MN (Figure 6 m).

## 4. Conclusion

We have shown for the first time that MN can be successfully prepared from hydrogel-forming materials. Such MN swell in skin to produce continuous, unblockable conduits from patch-type drug reservoirs to the dermal microcirculation, thus allowing prolonged transdermal drug administration. Pulsatile or bolus delivery can be achieved under electrical control, while MN are removed intact from skin. The ability of MN to imbibe skin interstitial fluid may find further use in minimally-invasive patient monitoring. MN materials were shown to be biocompatible and non-irritant in vitro and raised no safety concerns in human volunteers. This technology has the potential to overcome the limitations of conventional MN designs and greatly increase the range of type of drug deliverable transdermally, with ensuing benefits for industry, healthcare providers and, ultimately, patients. We are actively pursuing commercialisation of the technology with two major companies.
